# Medical Opinion and Movement

**Published:** 1910-12-03

**Authors:** 


### Medical Opinion and Movement.

### Papilloma of the Larynx.

The internal administration of calcined magnesia is recommended by Dr. Claoué, of Bordeaux, for the treatment of papillomata of the larynx. Although rare, the condition is nevertheless serious in children, especially on account of the ease with which the papillomata recur after removal. It appears that in veterinary medicine the efficacy of calcined magnesia against intrabuccal papillomata in dogs is already well established, and on this account Dr. Claoué was led to try it in two cases of laryngeal papillomata in children, with successful results. The treatment was carried on in both cases for some months, 50 centigrammes of the magnesia being administered daily for two or three weeks with intervals of the same duration. The condition was eventually completely cured in both cases. Dr. Sargnon, of Lyons, has also successfully treated a similar case in the same way, but in addition to internal administration he insufflated the magnesia upon the papillomata themselves.

### Œsophageal Stenosis.

In the *Zentralblatt für Chirurgie* Dr. H. Fischer reports an interesting case of stenosis of the œsophagus in an infant aged three years, in which a remarkable attempt was made to relieve the condition by means of a transthoracic operation. The operation was carried out by pulmonary insufflation according to the method devised by Meltzer and Auer, an account of which has already appeared in these columns. The child had swallowed some corrosive, resulting in an impassable stenosis of the œsophagus. Gastrostomy was performed, and a gastric fistula was established, but the patient did not thrive on this method of feeding, and was rapidly losing ground. All attempts at catheterising the stenosis in either direction failed, and transthoracic operation was decided upon. The eighth intercostal space was incised and the ribs drawn on one side, so as to render the diaphragm and œsophagus accessible. The lung was covered with compresses impregnated with sterilised vaseline and the posterior pleura incised over the œsophagus. The vagi were drawn aside and the œsophagus isolated and detached from the diaphragm at the level of its passage through. The peritoneum was then opened and 7 cm. of the upper end of the stomach drawn into the chest. The cardia was incised and a urethral sound passed through the stenosis up as far as the mouth, the lower end being drawn through the orifice of the gastrostomy. The gastric incision was then sutured and the operation completed. Everything went well until just at the end of the operation, when by a mistake some drops of liquid ether were allowed to enter the lung with the air. Immediate collapse followed, from which the child recovered, but died within twenty-four hours from pulmonary congestion. The result was disappointing, but the attempt is worthy of record as a piece of pioneer work in intrathoracic surgery.

### Subcutaneous Lymphatic Glands in Phthisis.

Dr. E. Gebrovsky draws attention to the frequent occurrence of palpable subcutaneous lymphatic glands on the lateral surface of the chest in patients suffering from tuberculosis of the pulmonary apices. Having observed these enlarged glands in two such cases, Dr. Gebrovsky undertook an examination of a large number of cases to determine the frequency of this sign. Altogether he examined 2,558 patients of varying ages from ten to seventy years; 929 of these showed well-marked signs of pulmonary tuberculosis, and of these 186, that is 20.02 per cent., had enlarged subcutaneous thoracic glands in the fourth and fifth intercostal spaces. Of the 1,629 remaining patients who showed no physical signs of pulmonary tuberculosis only 42 or 2.58 per cent. presented some enlargement of these glands. Fourteen of these were submitted to the ophthalmo-reaction and cuti-reaction with tuberculin, and all gave positive results. It was not possible to test the remaining twenty-eight in the same way, but several of them were suffering from some such affection as furuncles of the back or cancer of the œsophagus, which would explain the glandular enlargements. In the tuberculous patients the enlarged glands were generally on the same side as the pulmonary lesion, and the author submits that even if the glandular enlargement only occurs in 20 per cent. of the cases it nevertheless constitutes a sign of some clinical value.

### Paroxysmal Tachycardia.

In the *Livre Jubilaire du Professeur J. Tessier* Dr. G. Baccelli makes an interesting contribution in which he recommends the treatment of paroxysmal tachycardia by intravenous injection of strophanthine, and brings forward some important experimental and clinical evidence to support his views. He is of opinion that this morbid condition is not, as generally held, dependent upon any abnormal functioning of the vagi, but upon an irritative paralytic condition of the intracardiac ganglia. If the two vagi are exposed in a dog and an intravenous injection of strophanthine be made previous to division of these nerves, the injection is followed by a considerable elevation of blood-pressure and a slowing of the heart-beat. If the vagi are then divided the heart-beat is at once accelerated, but not to the same extent as is observed when the vagi are cut without any previous injection of strophanthine. Dr. Baccelli argues from this that the strophanthine acts upon the intracardiac ganglia, and through them upon the cardiac muscles. The author reports two cases of paroxysmal tachycardia with all the signs of failing heart, in which intravenous injections of strophanthine were most successful in their effects. The first patient was a man aged forty-eight, who had been for some time subject to attacks of paroxysmal tachycardia followed by serious symptoms of heart failure. The usual treatment with bromide, morphia, and subcutaneous injections of strophanthine was without effect, and the intravenous injections were then tried. Daily injections of strophanthine were administered, and each was followed by a sudden drop in the pulse-rate, an abundant diuresis, and a general improvement in the condition. At the end of a week the patient had returned to a normal condition, with a regular pulse of seventy to eighty beats a minute. The second patient had suffered from a similar attack of paroxysmal tachycardia for over a month, with the same severe symptoms of failing heart. The injections were followed by the same marked success, and a rapid disappearance of all the symptoms.

### Electricity for Gonorrhœal Rheumatism.

In an article in *La Tribune Médicale* Dr. P. C. Petit calls attention to the use of electricity in the treatment of gonorrhœal joints. The extreme pain caused by the slightest movement on the part of the patient causes him to keep the affected joint at rest. Wasting of the muscles is rapid, and there is an unfortunate tendency for the arthritis to go on to partial or complete ankylosis. It should therefore be the practitioner's aim to prevent this by the use of electricity. All that is required is a galvanic current administered through metal electrodes covered with absorbent lint. The poles should be applied so as to enclose the affected joint between them, but the exact point of application is of small importance. It is best to begin with a weak current, and to increase it gradually, as far as the patient can stand it, up to 40 milliampères. Each sitting should last for at least half an hour, and should be repeated twice a day. From the first there is relief from pain, and when the patient has recovered walking becomes easy, since the articulation is not implicated, and because the patient is less inclined to keep his limbs in a position favourable to the development of œdema. The vasomotor and trophic influence of the current hastens muscular resolution, and so cuts short the disease. As soon as pain has lessened, gentle movement of the articulation will further help to hasten recovery.

### Regulations for Colonists in Peru.

A Presidential resolution, dated June 17, 1910, has been issued through the Ministry of Fomento (Promotion) of the Peruvian Government, dealing with sanitary measures for the protection of the health of those who enter the country as colonists. The resolution states that in all contracts of companies or of individuals with the Government to establish colonies the following regulations must be complied with: (1) Colonists before embarking must be examined by a physician selected by the Peruvian Consul, who will give a health certificate which the Consul will legalise free of charge. (2) Those who have passed this examination must be vaccinated. (3) The Peruvian maritime sanitary regulations must be complied with during the passage from port of origin to Peru. (4) On arrival colonists will present their certificate to the medical inspector boarding the vessel. (5) The contractors are obliged to engage a physician and to supply medicines, as their contract declares. Articles 6 to 9 inclusive refer to the location of the colonists' houses on dry land, with an ample supply of running water near at hand, and with doors and windows properly protected by wire screens. (10) A hospital, also adequately screened, must be established in each colony, with a pharmacy containing the necessary remedies. (11) The physician of the colony will not only have charge of the sick, but also of the general hygiene of the community, the inspection of food and drink, and the prevention of infectious diseases, particularly those frequent in tropical regions. He must be supplied with the necessary medicines and apparatus, and also attend to the vaccination and revaccination of the colonists.

### Muhinyo—Malta Fever.

During the Uganda tour of the Sleeping Sickness Commission, Sir Apolo Kagwa (the Prime Minister) drew attention to an outbreak in the Ankole province of an alleged new disease named muhinyo. This condition had been regarded by one observer as allied to kala-azar, but no scientific study of its clinical features or its ætiology had been made. Eventually Sir D. Bruce went into the infected region and saw fifty cases of the disease, the average duration of which was found to be about three and a half months. No malarial or kala-azar parasites could be found in the patients; and as the clinical symptoms were those of a continued fever, agglutination reactions were sought to *Bacillus typhosus* and *Micrococcus melitensis.* To the latter test five out of seven patients gave a positive response; two of the five had their spleens punctured, and an organism was grown which was undoubtedly *melitensis.* A herd of goats was then secured from one of the villages in Ankole; three out of twenty-four agglutinated both the local muhinyo strain and one imported from Malta. From two of these goats the same organism was isolated, and Sir David regards it as established that muhinyo is Malta fever; and that in Uganda, as well as in Malta, it is transferred to man through the milk of infected goats.

### Japanese Treatment of Collapse.

In the *New York Medical Journal* Dr. Abrams describes *Kuatsu,* which is the Japanese term for the measures employed to resuscitate an individual suffering from a severe knock-out blow incurred in the national jiu-jitsu contests. The same method is also applied for any form of severe collapse after injuries, immersion, sunstroke, etc. The patient is put prone on the ground with the arms outspread. He is then pummelled vigorously over the seventh cervical vertebra many times in succession. The blows are repeated at short, regular intervals with the persistence of a carpenter driving a nail. When consciousness returns the patient is placed in a sitting posture, his arms are circumducted energetically, and he is then helped up and made to walk about. Otherwise, Dr. Abrams says, it is held that he may relapse into unconsciousness. The percussion of the vertebral column is best effected by a pneumatic hammer, though the fist is usually employed. It is supposed that these repeated concussions produce a reflex stimulation of the heart. The author has seen the method practised many times, and testifies to its extraordinary efficiency.

### The Production of Anæsthesia.

A new theory of the chemical physiology of anæsthesia is advanced by Dr. Buerker in the *Münch. Med. Wochenschrift.* It has long been supposed that the selective action of these drugs upon the nervous system may be accounted for by their great solubility in lipoids, which are present in greater proportion in nervous tissues than in any other. Such an accumulation does not suffice, according to this new hypothesis, though it is admitted to take a considerable share in the chemistry of narcosis. The author supposes that, besides this effect, narcotics also appropriate some of the active oxygen of the nerve tissues. The consequence is a temporary asphyxiation, which ends in paralysis of their physiological functions. The products of this oxidation of the narcotic explain in part the acidosis of anæsthesia, and account also for the increased formation of ammonia, which is required to neutralise the acids. Decomposed fats and lipoids are regarded as the sources of acetone, and the disturbances of metabolism thus set up are interpreted as the origin of the other after-effects of anæsthesia. This hypothesis has been elaborated largely from electrolytic findings and researches, and is summarised in the *Postgraduate*.

### Researches on Osteomalacia.

At one time the mysterious disease known as osteomalacia was treated by castration. Though this treatment has been abandoned on account of its failure, there is yet evidence of some subtle connection between the disease and the reproductive organs. Kownatski, in the *Münchener Medizin. Wochenschrift*, regards osteomalacia as a disease of failure of metabolism, and regards the ovary as having some share in provoking it. Accordingly he treats it either by giving the milk of castrated animals, which contains antibodies, or by administering the secretion of the suprarenal, which he believes to be an antagonistic gland. He records the case of a young woman so seriously affected as to be bedridden, and unable to move without great pain. He cured her with adrenalin hydrochloride. She recovered without deformity, and subsequently bore a normal child at term. Marquiz, writing on the same subject in *L'Obstétrique*, says that in pregnancy there is always a certain amount of decalcification, which is normal. An abnormal degree of this, with painful relaxation of the articulations, produces osteomalacia, and such extensive decalcification is accompanied by increased elimination of calcium in the urine. He believes that castration lessens this elimination, relieves symptoms, and improves the general condition. Decalcification is caused, he thinks, by ovarian, suprarenal, or other disturbances, the predisposing causes of which are too frequent pregnancies and malnutrition from insufficiency of food.

### Inversion of the Radial Reflex.

At a recent meeting of the Société Médicale des Hôpitaux, Dr. Babinski reported an alteration in the radial reflex which he has met with in a particular class of patients suffering from lesions of the cervical spinal cord. Normally percussion of the inferior end of the radius brings about a simple flexion of the forearm on the arm. When the tendon-reflexes of the upper extremity are well-marked, other movements, especially that of flexion of the fingers, may accompany flexion of the forearm. This is generally the case in patients suffering from hemiplegia of cerebral origin. In the healthy individual, however, the fingers alone are never seen to flex as the result of a radial shock. But this sort of transposition of the normal reflex movement, which the author proposes to call "inversion of the radial reflex," occurs sometimes in certain pathological circumstances. He has met with it in patients suffering from lesions of the cervical spinal cord, including syringomyelia and tumours, and up to the present has never noticed it in affections situated elsewhere. He believes that this inversion of a radial reflex is the result of a lesion affecting primarily the fifth cervical segment. From his observations he concludes that this sign by itself is sufficient to prove the existence of a lesion of the cervical spinal cord, and that it may help in the localisation of the injury.

### Combined Version during Pregnancy.

In the *American Journal of Obstetrics and Gynæcology* Dr. Stowe lays down certain rules in relation to fœtal malpresentations discovered before labour. His favourite method of correcting these is by a modification of Braxton Hicks's classical combined version; the essential difference is that, the cervix being intact, he makes no effort to pass two fingers through it as Braxton Hicks was wont to do when dealing with cases of actual labour. The proper presentation (which is not always a vertex in certain cases of contracted pelvis) should be decided and obtained before labour begins. It is often, however, impossible during the later weeks of pregnancy to correct a malpresentation by external version alone. Combined external and vaginal version is not dependent on effacement of the cervix; the less the cervix is dilated, the easier is the operation, when the membranes are intact. The dilatation of the perineum and vagina necessary in primiparæ often requires the assistance of an anæsthetic; but there is no tendency to termination of the pregnancy as a result of the manipulations. The danger of premature separation of the placenta is very slight; and the danger of septic infection of the uterus is reduced to a minimum because the hand does not enter it. The method can also be used during labour; if the membranes are intact it is quite simple, and there is less risk of rupturing the membranes than by Hicks's method. In placenta prævia, for instance, and other conditions, this is an advantage.

